# Motility of the Zoonotic Spirochete *Leptospira*: Insight into Association with Pathogenicity

**DOI:** 10.3390/ijms23031859

**Published:** 2022-02-07

**Authors:** Shuichi Nakamura

**Affiliations:** Department of Applied Physics, Graduate School of Engineering, Tohoku University, 6-6-05 Aoba, Aoba-ku, Sendai 980-8579, Japan; shuichi.nakamura.e8@tohoku.ac.jp; Tel.: +81-22-795-5849

**Keywords:** bacteria, motility, spirochete, *Leptospira*, leptospirosis, virulence factor, crawling, flagella

## Abstract

If a bacterium has motility, it will use the ability to survive and thrive. For many pathogenic species, their motilities are a crucial virulence factor. The form of motility varies among the species. Some use flagella for swimming in liquid, and others use the cell-surface machinery to move over solid surfaces. Spirochetes are distinguished from other bacterial species by their helical or flat wave morphology and periplasmic flagella (PFs). It is believed that the rotation of PFs beneath the outer membrane causes transformation or rolling of the cell body, propelling the spirochetes. Interestingly, some spirochetal species exhibit motility both in liquid and over surfaces, but it is not fully unveiled how the spirochete pathogenicity involves such amphibious motility. This review focuses on the causative agent of zoonosis leptospirosis and discusses the significance of their motility in liquid and on surfaces, called crawling, as a virulence factor.

## 1. Introduction

Many species of bacteria have motility operated with diverse mechanisms [1,2]. The flagellum is one of the major motility organs that are used by *Escherichia coli*, *Salmonella* spp., *Helicobacter pylori*, *Pseudomonas aeruginosa*, and others [3]. *Mycoplasma* spp. and *Myxococcus xanthus* move on surfaces, called gliding motility, and this is achieved via the molecular architecture on the cell surface [4,5]. *P. aeruginosa* and unicellular cyanobacteria also show motility on surfaces using extension and contraction of pili [6,7]. These bacteria rely on motility for navigation to explore preferred environments for growing, and pathogenic species utilize this ability for invading hosts [8,9].

Spirochetes are a group of Gram-negative bacteria and include pathogenic species, such as *Treponema pallidum* (syphilis), *Treponema denticola* (periodontal disease), *Brachyspira hyodysenteriae* (swine dysentery), *Borrelia burgdorferi* (Lyme disease), and *Leptospira interrogans* (leptospirosis). The spirochetes exhibit helical (e.g., *Leptospira* spp.) or flat-wave (e.g., *Borrelia* spp.) cell morphology and have multiple flagella within the periplasmic space. The spirochete flagella are called periplasmic flagella (PFs). The PF-dependent motility is known to be an essential virulence factor, but the mechanism of how spirochetes use the motility in the pathogenic process has not been fully understood. This review will focus on the two distinct modes of *Leptospira* motility and discuss their significance for pathogenicity.

## 2. Leptospirosis

Pathogenic leptospires colonize the proximal renal tubules of animals recovering from the disease, or reservoir hosts such as rodents. The bacteria are shed upon urination into environments, infecting animals in contact with the contaminated soil or water through injured skin. Diverse species of mammals have the potential to acquire *Leptospira* infection. Pathogenic *Leptospira* are classified into more than 300 serovars based on the structure of lipopolysaccharide (LPS), and the severity of the resultant symptom depends on the combination of *Leptospira* serovars and host species. In severe cases, the penetrating leptospires reach specific organs, such as the lung, liver, and kidney, causing hemorrhage, jaundice, and nephritis [10,11,12]. Animal experiments have shown that the loss-of-motility due to the knock-out of the PF-related genes reduces the virulence of *Leptospira*, suggesting that motility is an essential factor determining the pathogenicity [13,14].

## 3. Morphology and Motility of *Leptospira*

### 3.1. Cell Morphology

**Figure 1:**
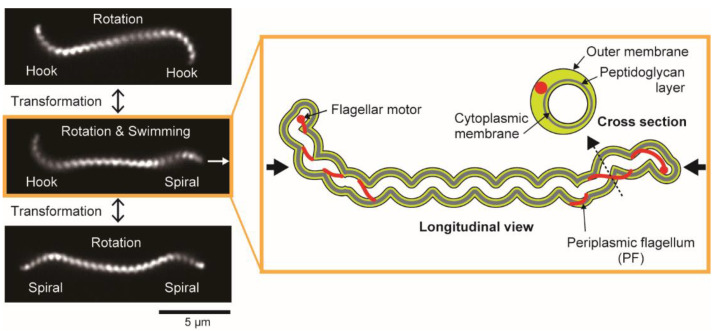
Morphology and motion forms of *Leptospira interrogans*. Dark-field micrographs (**left**) show three motion modes observed in the same cell with distinct cell-end morphologies. The cell rotates without net migration when exhibiting symmetric forms (Spiral–Spiral or Hook–Hook). Asymmetric form (Spiral–Hook) propels the cell in the direction indicated by the white arrow. Longitudinal and cross-sectional views of the asymmetric swimming mode are schematically depicted on the **right**. Thick black arrows indicate that the directions of cell-end gyration are defined by viewing the cellular tip from the cell exterior (see main text).

*Leptospira* spp. have two PFs (1 PF/cell end) within a thin (~150 nm in diameter), long (~20 μm in length), and short-pitch helical cell body (~700 nm in wavelength). The extracted PFs from the cell body exhibit a coiled shape, thus giving the cell ends curvature. The cell-end morphology depends on the gyration direction of the cell ends: Gyrating counterclockwise (CCW, viewing the cellular tip as indicated by thick black arrows in the cartoon of Figure 1) and clockwise (CW), the cell end form a “hook” shape and “spiral” shape, respectively (Figure 1) [15].

### 3.2. Swimming

Asymmetric configuration of the cell body propels the cell unidirectionally, and the anterior and posterior cell-body ends exhibit the spiral and hook shapes, respectively. The cell-end morphology frequently switches with the reversal of gyration, allowing the cell to change swimming direction. *Leptospira* often shows symmetric morphology (i.e., Spiral–Spiral or Hook–Hook), then rotating without net migration (Figure 1) [15,16]. Though the PF rotation has not been observed directly, its counter-torque is thought to turn the entire protoplasmic cylinder (PC). The combination of PC rotation (~100 Hz) and the spiral-end gyration (~50 Hz) produces thrust for swimming [17]. Motility assays showed that the migration distance by one revolution of PC is ~30% of the wavelength of the PC in a water-based solution, indicating that the swimming of *Leptospira* is a slippery motion [18]. However, in gel-like viscoelastic fluids (e.g., methylcellulose solution), the motion efficiency of *Leptospira* is improved up to 100% [15], resulting in an increment of the swimming velocity [19]. Interestingly, the addition of viscous agent to medium increases the frequency of swimming reversal, as discussed later [16,20].

### 3.3. Crawling

In 1975, Cox and Twigg showed a trail of the leptospiral movement on a smooth surface and called it “crawling” [21]. Charon et al. reported that unidentified outer membrane components have mobility along the cell body by observing the movement of microbeads attached to the cell surface via an anti-whole cell antibody [22]. Recently, Tahara et al. revealed that crawling is: (i) PF-dependent motility; (ii) conducted by only PC rotation without the direct contribution of the spiral end; (iii) mediated by adhesive mobile components residing in the outer membrane, such as lipopolysaccharide; and (iv) utterly slip-less motion [18]. Potential as a virulence factor of the *Leptospira* crawling will be discussed below.

## 4. Swimming in a Highly Viscous Milieu

### 4.1. Dependence on a Type of Polymer

The effect of viscosity on bacterial swimming has been investigated in many species [23], but we should note the type of polymers added to the media. For example, Ficoll, a highly branched polymer, makes a homogeneous viscous solution. In contrast, methylcellulose forms an elastic network in solution, and the bacterial movement in such a gel-like heterogeneous fluid depends on the size of the polymer network and bacteria [24,25]. Experiments and theoretical studies have shown that bacterial swimming in a gel-like fluid is accelerated monotonically or up to a certain point of the added polymer concentration, whereas the swimming speed decreases in Ficoll solutions [17,19,23,25,26,27,28]. The heterogeneous, viscoelastic milieu is ubiquitous in the host body (e.g., mucus layers covering tissues and extracellular matrix), implying the significance of swimming in such unique environments for pathogenicity.

### 4.2. Back-and-Forth Motion

**Figure 2:**
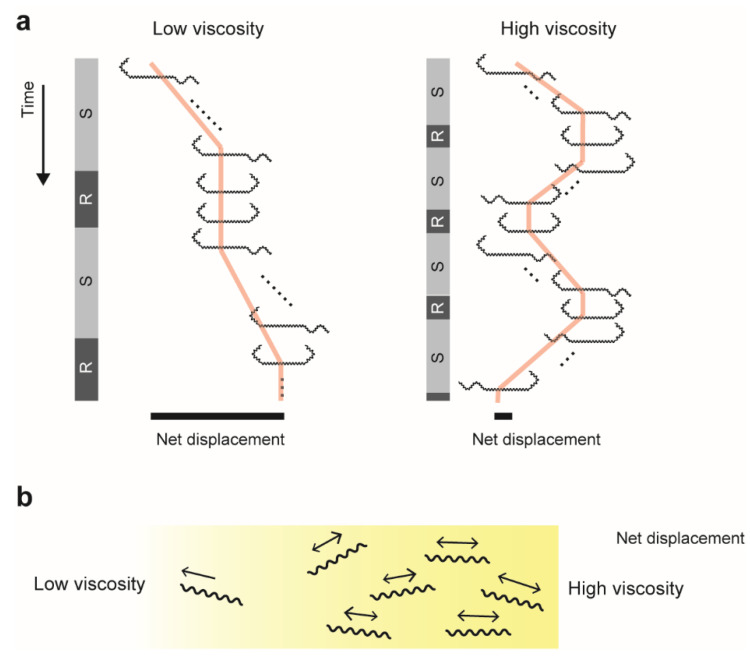
Enhancement of swimming reversal in high viscosity: (**a**) Schematic explanations of transition between swimming (S) and rotation (R) modes. For simplicity, the rotation mode indicates only Hook–Hook morphology. Time courses of cell positions show the effect of reversal on net displacement (black bars). (**b**) Accumulation of bacteria in high viscosity due to limitation of net migration. See [16] for more details.

In polymer solutions, *Leptospira* shows the speed variation in swimming [17,19,29] and increases the transition frequency between swimming (Spiral–Hook) and rotation (Spiral–Spiral and Hook–Hook) modes [16]. The enhancement of the motion-mode switching is observed in Ficoll, methylcellulose, and mucin solutions [16]. In addition, we revealed that the swimming direction reverses more frequently in high viscosity (Figure 2a) [16,20]. Enhancing “back and forth” movement suggests the limitation of the net migration, crowding bacteria within the mucus layer, and facilitating colonization over the tissues (Figure 2b).

### 4.3. Trial and Error?

**Figure 3:**
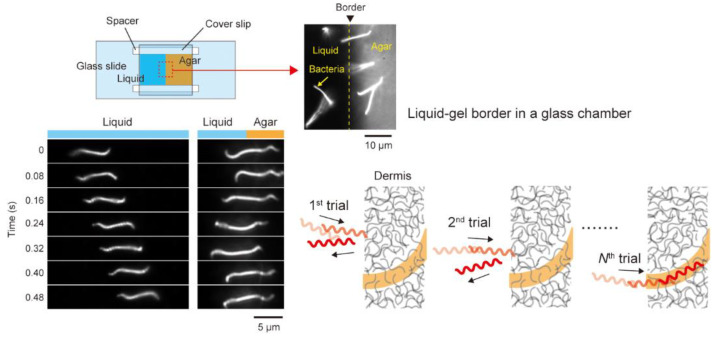
Swimming reversal enhanced in the liquid–gel border. The schematic (right bottom) explains a hypothetical scenario that the “trial-and-error”-like behavior allows the bacteria to find a path for easier invasion. This figure was made based on [20] with modifications.

The previous section describes the back-and-forth motion in viscous media, but the behavior is also observed at the liquid and gel interface (Figure 3) [20]. The mechanism sensing viscosity remains unknown, but the fact indicates that penetration of not the entire but partial cell body to a gel-like milieu allows *Leptospira* to change the swimming pattern. The experimental setup of the liquid–gel interface resembles wound skin exposed to environments contaminated by *Leptospira*. A time record of the *Leptospira* movement in the liquid–gel interface showed that the bacteria repeatedly attacked the interface while reversing the swimming direction and finally invading the gel phase (Figure 3). Swimming reversal induced in the liquid–gel interface could be interpreted as “trial-and-error” to search for an easier route for invasion.

### 4.4. Interaction between PFs

*Leptospira* switches swimming direction within <1 s [20]. Such a quick reversal suggests the coordination between two PFs mediated by unidentified signal transduction, but there is no definitive evidence so far. The most general signaling for the reversal of flagellar rotation involves the Che system: Sensing environmental stimuli via receptors induces phosphorylation (P) of the regulator protein CheY, and the binding of CheY-P to the flagellar motor reverses rotation. Noting that the diffusion constant (D) of CheY-P is ~10 μm^2^/s [30], the theoretical estimation using the formula $t=x^{2}/2D$, where t is the time for traveling the distance x with D, predicts the time gap of ~50 ms between the reversals of two flagellar motors in the same *E. coli* cell (x~1 μm). In contrast, since the distance between PFs of *Leptospira* is ~20 μm, the reversal upon CheY-P binding at one PF delays ~20 s from the other PF, indicating that the observed rapid reversal cannot be achieved only by the Che system. PFs of *Bo. burgdorferi* is so long that they overlap at the center of the cell body [31]. A theoretical study predicted that direct interaction between PFs is indispensable for propelling the *Bo. burgdorferi* smoothly [32]. In contrast, since PFs of *Leptospira* are too short for contact with each other directly, the cell body might mediate the interaction between PFs [17,33]. There remains the possibility that *Leptospira* switches the PF rotation and transforms the cell end (Spiral or Hook shape) at random, and the asymmetric swimming mode (Spiral–Hook) appears accidentally. However, the simple scenario will not explain the viscosity dependence of the swimming reversal [16,20]. Higher-resolution analysis focusing on the reversal timing of two PFs (cell-end gyration) may give a clue to unveil this long-term mystery.

## 5. Swimming Force

**Figure 4:**
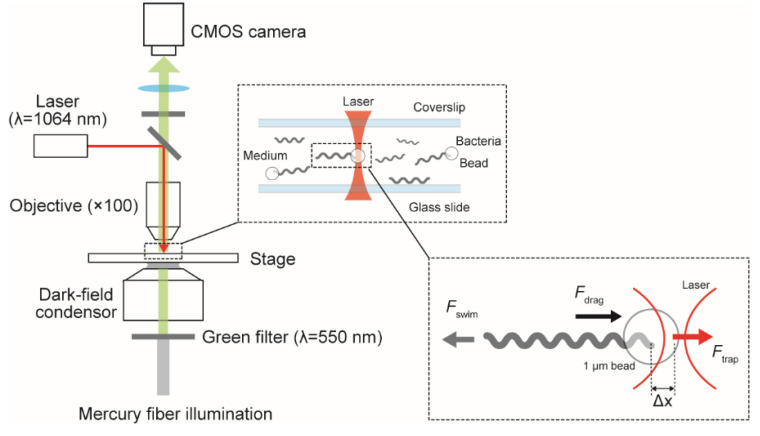
Measurement of swimming speed using optical tweezers. A microbead (1 μm in diameter) attached to a swimming bacterium is trapped by a laser. Swimming force ($F_{\mathrm{swim}}$) is determined from the bead displacement (Δx), drag force exerted on the bacterium ($F_{\mathrm{drag}}$), and trap force ($F_{\mathrm{trap}}$) [20].

There are many parameters to characterize bacterial motility. For example, swimming speed (*v*) and the rotation speed of flagella (*f*) have been measured in various bacterial species. The theoretical estimation of drag coefficients for translation ($\gamma_{T}$) and rotation ($\gamma_{R}$) allows us to calculate swimming force ($F=\gamma_{T}v$) and torque generated by the flagellar motor ($N=\gamma_{R}2\pi f$). Direct measurement of force and torque is not as easy as speed, but some physical techniques, such as optical tweezers [34] and the electrorotation method [35], have achieved the experiment. Since adequate power seemed to be needed for *Leptospira* to invade the host body through the dermis, we measured the swimming force using optical tweezers. A focused laser can trap a micro-object at the focusing point, thereby manipulating the trapped object by moving the laser position. Moreover, when trapping mobile objects such as microorganisms, the optical tweezers impose the restoring force on the trapped object in a displacement-dependent manner. Therefore, the optical tweezers can be used as a spring scale (Figure 4). Trapping a microbead attached to the surface of the *Leptospira* cell revealed that the spirochete generates ~17 pN by swimming [20], which is about 30 times the swimming force of *E. coli* [34]. *Leptospira* might be a powerful swimmer. The PFs of *Leptospira* are fueled by proton motive force [36], just like with the *E. coli* motor, and perhaps there is not be much difference in the amount between the species. Feasible explanations of the powerful swimming of *Leptospira* are as follows. First, cryo-electron microscopy revealed that the rotor ring of the *Leptospira* flagellar motor is larger than that of the *E. coli* motor, therefore generating higher torque [37]. Secondarily, drag coefficients for the *Leptospira* cell body are greater than the *E. coli* one due to its very long cell body [20].

## 6. Association of Crawling with Pathogenicity

### 6.1. Crawling on Cultured Kidney Cells

**Figure 5:**
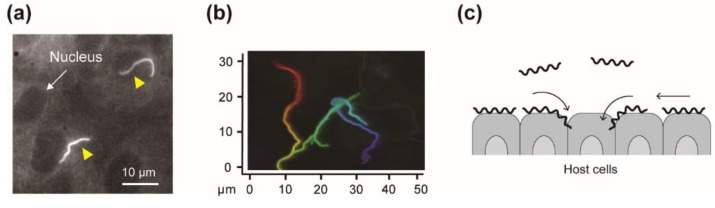
Crawling of *Leptospira* on the cultured kidney cells [38]: (**a**) Epi-fluorescence image of the *L. interrogans* serovar Icterohaemorrhagiae on the rat kidney cell line (NRK-52E). (**b**) A trajectory of a crawling *L. interrogans*; time courses in the order of red, orange, yellow, green, blue, and indigo. (**c**) A hypothesis of crawling-dependent pathogenicity. This figure was made using some data reported in [38] with some modifications.

The swimming motility would be somehow associated with the pathogenicity of *Leptospira*, but the bacterial adhesion to, and movement over tissue surfaces could be related to manifestation more directly. Recently, we assessed the leptospiral dynamics on the cultured kidney cells derived from various mammalian species (Figure 5a) [38]. Comparison of the bacterial adhesion to kidney-cell sheets between pathogenic and non-pathogenic strains showed higher adhesivity of the pathogenic strains. There was no difference in crawling speed among the measured strains, but pathogens’ crawling showed wider spreading over the kidney cells (Figure 5b). An in vitro infection assay using renal proximal tubule epithelial cells (RPTECs) showed that the adhesion and crawling populations of the pathogenic species *L. interrogans* on RPTECs increased with time (~24 h after infection). In contrast, those of non-pathogenic species *Leptospira biflexa* were smaller than *L. interrogans* without changing during the experiment [39]. The adhesivity and crawling could allow *Leptospira* to interact with the tissue surfaces, increasing the probability of reaching an intracellular tight junction, a transmigration route for *Leptospira*, and assisting disassembly of junction complexes (Figure 5c).

### 6.2. Leptospira Ligands Involved in Crawling

Anti-LPS antibody affects crawling on a glass surface, suggesting a potential role of LPS as an adhesin in surface motility [18]. *Leptospira* have abundant cell-surface proteins, and some of them are known to bind to components included in the extracellular matrix, such as fibronectin and laminin [11,40]. For example, Lig (leptospiral immunoglobulin-like protein) has a binding affinity to fibronectin, laminin, and collagen [41,42]; LenA (leptospiral endostatin-like protein A) binds to the complement negative regulator Factor H and laminin [43,44,45]. Future studies will address the responsibility of these candidate proteins for the *Leptospira* dynamics on the host cells.

### 6.3. Host Preference

The severity of leptospirosis depends on the combination of the host species and the *Leptospira* serovars, but the link between the host–pathogen pairing and the outcome is ambiguous. Though it would be the consequence of a complicated host–pathogen interaction, such as an immunological attack and evasion [46], which might lead us to understand the mechanism from the *Leptospira* dynamics on the host tissues. The adhesivity and crawling-dependent spread investigated on kidney cells of various mammalian hosts show a positive correlation, and the typical maintenance host rat shows a lower score against pathogenic strains (i.e., low adhesivity and limited spread) [38]. In contrast, the pairs have a trend causing severe symptoms (e.g., *L. interrogans* serovar Manilae vs. human and Icterohaemorrhagiae vs. dogs), and show a higher score [38]. The tendency plausibly suggests the significance of the pathogens’ motility on the host tissues for leptospirosis (Figure 5c).

## 7. Conclusions

This review article summarized the current knowledge on *Leptospira* motility while focusing on its association with pathogenicity. It emphasized the significance of motility as a virulence factor; however, most proposals on motility-dependent pathogenicity remain a matter of speculation. If future works prove the role of motility more definitively, motility could be a novel target for medication and prevention of infection. For example, adhesive outer-membrane components, which could be involved in crawling [38], might be new antimicrobial targets. Recent results have highlighted the significance of motility over solid surfaces. *T. denticola* also shows crawling motility using some outer-membrane components such as the surface proteinase dentilisin, facilitating the surface spreading of the spirochete [47]. A study of *Bo. burgdorferi* reported multiple forms of motility on gelatin, which are perhaps different from crawling, and discussed their relevance to the spirochete dissemination on the host tissue [48]. Direct interaction with host cells would be crucial for the pathogenic process. Therefore, not limited to spirochetes, further intensive investigation of the pathogens’ dynamics on the host cells is expected.

## References

[1] Miyata M., Robinson R.C., Uyeda T.Q.P., Fukumori Y., Fukushima S., Haruta S., Homma M., Inaba K., Ito M., Kaito C. (2020). Tree of Motility—A Proposed History of Motility Systems in the Tree of Life. Genes Cells.

[2] Jarrell K.F., McBride M.J. (2008). The Surprisingly Diverse Ways That Prokaryotes Move. Nat. Rev. Microbiol..

[3] Nakamura S., Minamino T. (2019). Flagella-Driven Motility of Bacteria. Biomolecules.

[4] Miyata M. (2010). Unique Centipede Mechanism of *Mycoplasma* Gliding. Annu. Rev. Microbiol..

[5] Faure L.M., Fiche J.-B., Espinosa L., Ducret A., Anantharaman V., Luciano J., Lhospice S., Islam S.T., Tréguier J., Sotes M. (2016). The Mechanism of Force Transmission at Bacterial Focal Adhesion Complexes. Nature.

[6] Burrows L.L. (2012). *Pseudomonas aeruginosa* Twitching Motility: Type IV Pili in Action. Annu. Rev. Microbiol..

[7] Wilde A., Mullineaux C.W. (2015). Motility in Cyanobacteria: Polysaccharide Tracks and Type IV Pilus Motors. Mol. Microbiol..

[8] Haiko J., Westerlund-Wikström B. (2013). The Role of the Bacterial Flagellum in Adhesion and Virulence. Biology.

[9] Josenhans C., Suerbaum S. (2002). The Role of Motility as a Virulence Factor in Bacteria. Int. J. Med. Microbiol..

[10] Adler B., de la Peña Moctezuma A. (2010). *Leptospira* and Leptospirosis. Vet. Microbiol..

[11] Picardeau M. (2017). Virulence of the Zoonotic Agent of Leptospirosis: Still Terra Incognita?. Nat. Rev. Microbiol..

[12] Coburn J., Picardeau M., Woods C.W., Veldman T., Haake D.A. (2021). Pathogenesis Insights from an Ancient and Ubiquitous Spirochete. PLoS Pathog..

[13] Lambert A., Picardeau M., Haake D.A., Sermswan R.W., Srikram A., Adler B., Murray G.A. (2012). FlaA Proteins in *Leptospira Interrogans* Are Essential for Motility and Virulence but Are Not Required for Formation of the Flagellum Sheath. Infect. Immun..

[14] Wunder E.A., Figueira C.P., Benaroudj N., Hu B., Tong B.A., Trajtenberg F., Liu J., Reis M.G., Charon N.W., Buschiazzo A. (2016). A Novel Flagellar Sheath Protein, FcpA, Determines Filament Coiling, Translational Motility and Virulence for the *Leptospira* Spirochete. Mol. Microbiol..

[15] Goldstein S.F., Charon N.W. (1990). Multiple-Exposure Photographic Analysis of a Motile Spirochete. Proc. Natl. Acad. Sci. USA.

[16] Takabe K., Tahara H., Islam M.S., Affroze S., Kudo S., Nakamura S. (2017). Viscosity-Dependent Variations in the Cell Shape and Swimming Manner of *Leptospira*. Microbiology.

[17] Nakamura S., Leshansky A., Magariyama Y., Namba K., Kudo S. (2014). Direct Measurement of Helical Cell Motion of the Spirochete *Leptospira*. Biophys. J..

[18] Tahara H., Takabe K., Sasaki Y., Kasuga K., Kawamoto A., Koizumi N., Nakamura S. (2018). The Mechanism of Two-Phase Motility in the Spirochete *Leptospira*: Swimming and Crawling. Sci. Adv..

[19] Takabe K., Nakamura S., Ashihara M., Kudo S. (2013). Effect of Osmolarity and Viscosity on the Motility of Pathogenic and Saprophytic *Leptospira*. Microbiol. Immunol..

[20] Abe K., Kuribayashi T., Takabe K., Nakamura S. (2020). Implications of Back-and-Forth Motion and Powerful Propulsion for Spirochetal Invasion. Sci. Rep..

[21] Cox P.J., Twigg G.I. (1974). Leptospiral Motility. Nature.

[22] Charon N.W., Lawrence C.W., O’Brien S. (1981). Movement of Antibody-Coated Latex Beads Attached to the Spirochete *Leptospira Interrogans*. Proc. Natl. Acad. Sci. USA.

[23] Schneider W.R., Doetsch R.N. (1974). Effect of Viscosity on Bacterial Motility. J. Bacteriol..

[24] Berg H.C., Turner L. (1979). Movement of Microorganisms in Viscous Environments. Nature.

[25] Magariyama Y., Kudo S. (2002). A Mathematical Explanation of an Increase in Bacterial Swimming Speed with Viscosity in Linear-Polymer Solutions. Biophys. J..

[26] Nakamura S., Adachi Y., Goto T., Magariyama Y. (2006). Improvement in Motion Efficiency of the Spirochete *Brachyspira Pilosicoli* in Viscous Environments. Biophys. J..

[27] Ruby J.D., Charon N.W. (1998). Effect of Temperature and Viscosity on the Motility of the Spirochete *Treponema Denticola*. FEMS Microbiol. Lett..

[28] Kimsey R.B., Spielman A. (1990). Motility of Lyme Disease Spirochetes in Fluids as Viscous as the Extracellular Matrix. J. Infect. Dis..

[29] Kaiser G.E., Doetsch R.N. (1975). Enhanced Translational Motion of *Leptospira* in Viscous Environments. Nature.

[30] Terasawa S., Fukuoka H., Inoue Y., Sagawa T., Takahashi H., Ishijima A. (2011). Coordinated Reversal of Flagellar Motors on a Single *Escherichia Coli* Cell. Biophys. J..

[31] Charon N.W., Cockburn A., Li C., Liu J., Miller K.A., Miller M.R., Motaleb M.A., Wolgemuth C.W. (2012). The Unique Paradigm of Spirochete Motility and Chemotaxis. Annu. Rev. Microbiol..

[32] Vig D.K., Wolgemuth C.W. (2012). Swimming Dynamics of the Lyme Disease Spirochete. Phys. Rev. Lett..

[33] Takabe K., Kawamoto A., Tahara H., Kudo S., Nakamura S. (2017). Implications of Coordinated Cell-Body Rotations for *Leptospira* Motility. Biochem. Biophys. Res. Commun..

[34] Chattopadhyay S., Moldovan R., Yeung C., Wu X.L. (2006). Swimming Efficiency of Bacterium *Escherichia Coli*. Proc. Natl. Acad. Sci. USA.

[35] Sato K., Nakamura S., Kudo S., Toyabe S. (2019). Evaluation of the Duty Ratio of the Bacterial Flagellar Motor by Dynamic Load Control. Biophys. J..

[36] Islam M.S., Morimoto Y.V., Kudo S., Nakamura S. (2015). H^+^ and Na^+^ Are Involved in Flagellar Rotation of the Spirochete *Leptospira*. Biochem. Biophys. Res. Commun..

[37] Beeby M., Ribardo D.A., Brennan C.A., Ruby E.G., Jensen G.J., Hendrixson D.R. (2016). Diverse High-Torque Bacterial Flagellar Motors Assemble Wider Stator Rings Using a Conserved Protein Scaffold. Proc. Natl. Acad. Sci. USA.

[38] Xu J., Koizumi N., Nakamura S. (2020). Crawling Motility on the Host Tissue Surfaces Is Associated with the Pathogenicity of the Zoonotic Spirochete *Leptospira*. Front. Microbiol..

[39] Sebastián I., Okura N., Humbel B.M., Xu J., Hermawan I., Matsuura C., Hall M., Takayama C., Yamashiro T., Nakamura S. (2021). Disassembly of the Apical Junctional Complex during the Transmigration of *Leptospira Interrogans* across Polarized Renal Proximal Tubule Epithelial Cells. Cell. Microbiol..

[40] Haake D.A., Matsunaga J. (2010). Leptospira: A Spirochaete with a Hybrid Outer Membrane. Mol. Microbiol..

[41] Matsunaga J., Barocchi M.A., Croda J., Young T.A., Sanchez Y., Siqueira I., Bolin C.A., Reis M.G., Riley L.W., Haake D.A. (2003). Pathogenic *Leptospira* Species Express Surface-Exposed Proteins Belonging to the Bacterial Immunoglobulin Superfamily. Mol. Microbiol..

[42] Choy H.A., Kelley M.M., Chen T.L., Møller A.K., Matsunaga J., Haake D.A. (2007). Physiological Osmotic Induction of *Leptospira Interrogans* Adhesion: LigA and LigB Bind Extracellular Matrix Proteins and Fibrinogen. Infect. Immun..

[43] Stevenson B., Choy H.A., Pinne M., Rotondi M.L., Miller M.C., DeMoll E., Kraiczy P., Cooley A.E., Creamer T.P., Suchard M.A. (2007). *Leptospira Interrogans* Endostatin-like Outer Membrane Proteins Bind Host Fibronectin, Laminin and Regulators of Complement. PLoS ONE.

[44] Verma A., Hellwage J., Artiushin S., Zipfel P.F., Kraiczy P., Timoney J.F., Stevenson B. (2006). LfhA, a Novel Factor H-Binding Protein of *Leptospira Interrogans*. Infect. Immun..

[45] Barbosa A.S., Abreu P.A.E., Neves F.O., Atzingen M.V., Watanabe M.M., Vieira M.L., Morais Z.M., Vasconcellos S.A., Nascimento A.L.T.O. (2006). A Newly Identified Leptospiral Adhesin Mediates Attachment to Laminin. Infect. Immun..

[46] Daroz B.B., Fernandes L.G.V., Cavenague M.F., Kochi L.T., Passalia F.J., Takahashi M.B., Nascimento Filho E.G., Teixeira A.F., Nascimento A.L.T.O. (2021). A Review on Host-*Leptospira* Interactions: What We Know and Future Expectations. Front. Cell. Infect. Microbiol..

[47] Kokubu E., Kikuchi Y., Okamoto-Shibayama K., Nakamura S., Ishihara K. (2021). Crawling Motility of *Treponema*
*denticola* Modulated by Outer Sheath Protein. Microbiol. Immunol..

[48] Harman M.W., Dunham-Ems S.M., Caimano M.J., Belperron A.A., Bockenstedt L.K., Fu H.C., Radolf J.D., Wolgemuth C.W. (2012). The Heterogeneous Motility of the Lyme Disease Spirochete in Gelatin Mimics Dissemination through Tissue. Proc. Natl. Acad. Sci. USA.

